# The Lifelong Health Support 10: a Japanese prescription for a long and healthy life

**DOI:** 10.1265/ehpm.22-00085

**Published:** 2022-06-09

**Authors:** Ahmed Arafa, Yoshihiro Kokubo, Rena Kashima, Masayuki Teramoto, Yukie Sakai, Saya Nosaka, Youko M. Nakao, Emi Watanabe

**Affiliations:** 1 Department of Preventive Cardiology, National Cerebral and Cardiovascular Center, Suita, Japan; 2 Department of Public Health, Faculty of Medicine, Beni-Suef University, Beni-Suef, Egypt; 3 Leeds Institute of Cardiovascular and Metabolic Medicine, University of Leeds, Leeds, UK; 4 Department of Food and Nutrition, Faculty of Contemporary Human Life Science, Tezukayama University, Nara, Japan

**Keywords:** Lifelong Health Support 10, Cancer, Cardiovascular diseases, Lifestyle, Japan

## Abstract

**Background:**

Although the age-adjusted incidence and mortality of cancer and cardiovascular disease (CVD) have been decreasing steadily in Japan, both diseases remain major contributors to morbidity and mortality along with the aging society. Herein, we aim to provide a prescription of 10 health tips for long and healthy life named the “Lifelong Health Support 10 (LHS10).”

**Method:**

The LHS10 was developed by the preventive medicine specialists at the National Cerebral and Cardiovascular Center in Suita, where it has been used for health guidance to prevent CVD, cancer, and cognitive decline in addition to their major risk factors such as hypertension, diabetes, and obesity. It consisted of the lifestyle modification recommendations of the 2014 Japanese Society of Hypertension guidelines and the 2017 Japan Atherosclerosis Society Guidelines for preventing atherosclerotic CVD. Further, it came in line with other international lifestyle modification guidelines. In this narrative review, we summarized the results of several Japanese epidemiological studies investigating the association between the LHS10 items and the risk of cancer, CVD, and other chronic diseases including dementia, diabetes, and chronic kidney disease.

**Results:**

The LHS10 included avoiding smoking and secondhand smoke exposure, engaging in physical activity, refraining from excessive alcohol drinking, reducing fried foods and sugary soft drinks, cutting salt in food, consuming more vegetables, fruits, fish, soy foods, and fibers, and maintaining proper body weight. All items of the LHS10 were shown to reduce the risk of cancer, CVD, and other chronic diseases.

**Conclusions:**

The LHS10 can be a helpful tool for health guidance.

**Supplementary information:**

The online version contains supplementary material available at https://doi.org/10.1265/ehpm.22-00085.

## Graphical Abstract

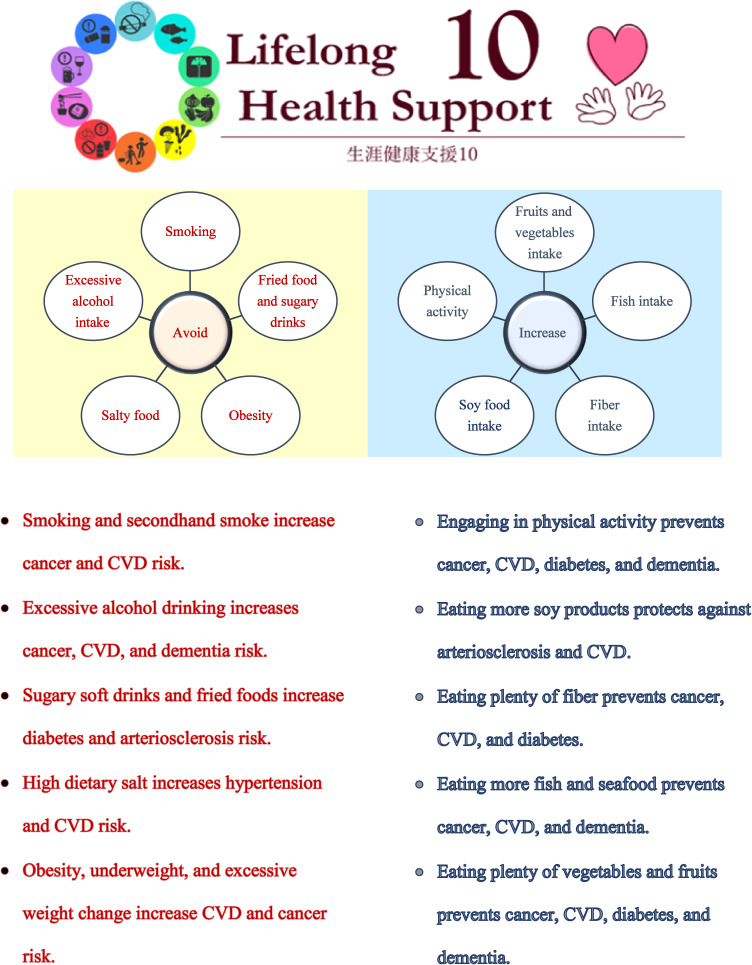

## Introduction

The previous decades have witnessed a Japanese public health miracle with sustained reductions in age-adjusted cancer and cardiovascular disease (CVD) mortality rates. These reductions were associated with concomitant increases in the average and healthy life expectancies to reach, in 2019, 81.5 and 72.6 years in men and 86.9 and 75.5 years in women [1]. Such a miracle would not have happened without controlling the modifiable risk factors for cancer and CVD [2, 3]. To further promote people’s health, the Japanese government initiated the “Health Japan 21” project. This project involved a long-term plan aiming at extending healthy life expectancy, reducing health disparities, expanding social welfare services, and improving the social environment by implementing policies and health promotion programs to improve dietary habits, encourage physical activity (PA), and reduce smoking and excessive alcohol drinking [4].

Primary prevention of morbidities and mortalities related to chronic diseases, especially cancer and CVD, necessitates health promotion and education using applicable concise health tips to improve the daily lifestyle. To reach this goal, preventive medicine specialists, including physicians, nurses, and dietitians, at the National Cerebral and Cardiovascular Center in Suita have developed in 2019, the “Lifelong Health Support 10 (LHS10),” a health guidance tool of 10 health items, inspired by the Japanese lifestyle, that allows for assessing different lifestyle aspects. The items of the LHS10 were a combination of the 2014 Japanese Society of Hypertension (JSH) lifestyle modification guidelines [5] and the 2017 Japan Atherosclerosis Society (JAS) guidelines for preventing atherosclerotic CVD [6]. A total of 7 health tips were extracted from the JSH 2014 guidelines: smoking cessation, performing PA, avoiding excessive drinking, healthy diet (reducing salt, increasing the intake of fruits, vegetables, and fish), and maintaining appropriate body weight. The JAS 2017 guidelines included, in addition to the previous items, other items, such as avoiding fried food and sugary soft drink and consuming more fibers and soy products. Similar items were suggested by 6 Japanese highly specialized medical research centers to prevent cancer [7]. Other Japanese literature showed that many LHS10 items could also protect from cognitive decline [8]. Importantly, these items came in line with several international lifestyle modification guidelines to protect from cancer and CVD [9] (Supplemental figure 1).

Many Japanese lifestyle modification guidelines were extracted from studies conducted on Western populations [7–9] who carry different sociodemographic, personal, and medical characteristics. We, therefore, described, in this narrative review, the current Japanese epidemiological evidence of the preventive effect of the LHS10 items against the risk of cancer, CVD, and other chronic diseases, such as diabetes and dementia, among the Japanese and provided concise health tips for a long and healthy life.

## Components of Lifelong Health Support 10

### 1. Avoid smoking

The prevalence of smoking in Japan has been decreasing among adolescents, middle-aged, and older adults of both genders, accompanied by limiting access of minors to cigarettes, prohibiting smoking in the workplace, providing smoking prevention and quitting programs, and putting a high tax on cigarettes [10, 11]. In 2018, the crude and age-adjusted prevalence rates of current smoking among people aged ≥15 years in Japan reached their lowest levels in decades: 31.1% and 33.2% in men and 8.6% and 10.5% in women, respectively [12].

The negative health impacts of smoking are enormous. The 6-prefecture Study showed that smoking strikingly increased cancer mortality in Japanese men and women: lung cancer (350% and 150%), esophagus cancer (120% and 70%), liver cancer (50% and 60%), pancreatic cancer (50% and 60%), stomach cancer (50% and 20%), and urinary bladder cancer (70% and 90%) [13]. Later studies using data from large population-based cohort studies including the National Integrated Project for Prospective Observation of Non-communicable Disease And its Trends in the Aged (NIPPON DATA80) [14], the Japan Public Health Center-based Prospective Study (JPHC Study) [15, 16], and the Japan Collaborative Cohort Study (JACC Study) [17], reached the same conclusion.

A pooled analysis of 3 large prospective cohort studies showed that smoking was associated with increased CVD, coronary heart disease (CHD), and stroke mortality by 51%, 119%, and 24% in men and 85%, 148%, and 70% in women, respectively [18]. Alike the Suita Study showed increased CVD mortality among smokers and the hazardous effect of smoking was augmented among those with metabolic syndrome [19]. Besides, smoking is an essential component of most Japanese CHD, stroke, atrial fibrillation (AF), and hypertension risk scores [20–27]. Furthermore, a recent study pooling data from 9 Japanese population-based cohort studies showed that even low-dose intensity smoking increased all-cause, cancer, and CVD mortality among men and women with a dose-response manner [28].

Apart from cancer and CVD risk, midlife smoking, in the Hisayama Study, more than doubled the risk of dementia among older adults [29]. A meta-analysis of 22 Japanese studies showed that smoking increased the risk of diabetes by 38% [30]. In addition, 2 cohort studies including residents and workers from Nagasaki and Ishikawa prefectures showed increased chronic kidney disease (CKD) risk among smokers [31, 32].

Fortunately, the mortality risk due to CVD, CHD, and stroke could decrease within 5 years of smoking cessation to reach the same level of never smokers after 10 years [18, 33]. A pooled analysis of 3 cohort studies showed that compared to current smokers who started smoking at their 20s of age, former smokers who started smoking at the same age as current smokers but quit before their 70s of age had a lower risk of lung cancer mortality, and those who quit earlier seized greater benefits than those who quit later [34]. Smoking cessation for 3 years was enough to cut dementia risk to the level of never smokers [35] and smoking cessation for 5 years reduced the risk of diabetes to the normal level [30].

Secondhand smoke exposure is also a risk factor for cancer incidence and mortality. A meta-analysis of 9 Japanese epidemiological studies showed that exposure to secondhand smoke was associated with a 28% increase in lung cancer incidence [36]. In the JACC Study, never smokers who had ≥3 smoking family members during childhood showed a 132% increase in pancreatic cancer mortality during adulthood [37]. The 3-Prefecture Cohort Study indicated that secondhand smoke exposure was associated with stroke mortality in women [38]. Besides, the use of e-cigarettes and heat-not-burn tobacco has been increasing in Japan, especially among young adults. The vaping process could evolve chemicals with carcinogenic properties, making this behavior a rising public health challenge [39, 40].

Our first health tip is to avoid smoking and secondhand smoke exposure, and for current smokers is to quit smoking. It is understood that quitting smoking or even cutting down the number of cigarettes is not an easy decision. However, it could be achieved by consulting health specialists for nicotine replacement therapy such as Varenicline which reduces cravings for nicotine and blocks the rewarding and reinforcing effects of smoking. Other methods including getting support from family, making a non-smoking environment, engaging in PA, practicing relaxation techniques such as deep-breathing exercises and yoga, and chewing sugarless gums could be helpful. Unlike the belief that e-cigarettes can help quit smoking, one study including Japanese people aged 20–69 years showed that e-cigarette use was negatively associated with smoking cessation, while replacement therapy was positively associated with smoking cessation [41]. On the country level, the World Health Organization (WHO) introduced the MPOWER measures that included Monitoring tobacco use, Protecting people from smoking, Offering help to quit smoking, Warning about the dangers of smoking, Enforcing tobacco advertising, promotion, and sponsorship bans, and Raising taxes on cigarettes [42].

### 2. Engage in physical activity

Given the variations in PA definitions and assessment procedures, determining the prevalence and trends of PA engagement in Japan is challenging. For example, 20% of schoolchildren in rural areas met the WHO criteria for PA by practicing moderate to vigorous PA ≥60 minutes/day [43], while 74.7% of boys and 55.2% of girls in urban schools met the Japan Sports Association recommendations by practicing PA ≥7 hours/week [44]. The Hisayama Study showed that community-dwelling adults aged ≥40 years practiced moderate to vigorous PA for an average of 54.4 minutes/day and 34.8% of participants practiced moderate to vigorous PA ≥150 minutes/week in bouts ≥10 minutes [45]. According to the National Health and Nutrition Survey, 36% of men and 29% of women in Japan practiced PA ≥30 minutes twice/week during 2017, and this prevalence did not change during the preceding decade [46].

The health benefits of PA are well-documented. The JPHC Study showed that men and women in the highest PA quartile showed lower cancer risk by 13% and 16% [16]. A dose-response relationship between engaging in PA and the reduced risk of CVD and stroke was also seen in the JPHC Study [47]. The JACC Study revealed that compared to walking 0.5 hours/day, walking ≥1 hour/day was associated with a 16% reduction in CVD mortality, and practicing sport ≥5 hours/week compared to 1–2 hours/week reduced CVD mortality by 27% [48]. The Jichi Medical School (JMS) cohort study showed that PA reduced CVD mortality in people with and without a history of CVD [49, 50]. Frequent stair climbing was associated with a reduced risk of AF in the Suita Study [51].

In addition to protecting from cancer and CVD, the Japan Gerontological Evaluation Study (JAGES) and the Hisayama Study showed that PA was associated with a lower risk of dementia among older adults, and the increasing duration and intensity of PA augmented the protective effect [52–55]. The Japan Epidemiology Collaboration on Occupational Health (J-ECOH) Study indicated that vigorous PA could prevent depressive symptoms [56]. Even engaging in PA once/week was associated with a reduced risk of diabetes in the Osaka Health Survey [57].

Our second health tip is to get engaged in PA. This engagement should be gradual in duration and intensity, but consistent. Walking and stretching could be a good start for physically inactive people who do not have the required time or equipment to practice PA. Increasing the duration and intensity of PA overtime could effectively promote health. Practicing PA in groups or with friends endures its continuity. Other readily available forms of PA such as climbing stairs instead of using elevators or escalators, going to work on foot or by bicycle whenever possible, and increasing time for housework should be considered as well. According to the WHO and the Ministry of Health, Labor and Welfare of Japan, adults (20–64 years) should engage in ≥60 minutes/day of moderate-to-vigorous PA and older adults (≥65 years) should engage in ≥40 min/day of PA at any intensity [43, 44].

### 3. Refrain from excessive drinking

According to the Periodical Nationwide Surveys, the prevalence of Japanese alcohol drinkers did not significantly change between 2003 and 2013: 83.6% and 82.9% in men versus 72.3% and 72.0% in women [58]. The good sign was that during the same period, daily drinkers decreased from 36.8% to 29.4% in men and from 22.1% to 18.4% in women, and alcohol dependence decreased from 5.8% to 4.2% in men and from 3.6% to 2.7% in women. The bad sign was that the age of first-time drinking and excessive drinking declined in both sexes [58]. Besides, the National Death Registry showed that alcohol-related deaths increased from 4.0 to 5.2/100,000 between 1995 and 2013 before they decreased to 5.0/100,000 in 2016. Reducing alcohol taxes and increasing alcohol sales were associated with higher alcohol-related deaths [59].

A J-shaped association between alcohol consumption and mortality was described in Japanese literature. The JPHC Study, for example, concluded that while occasional and light to moderate drinkers showed a lower risk of mortality from cancer and CVD than never-drinkers, excessive drinkers had a higher risk of cancer and CVD mortality [60, 61], and cancer risk was higher among smokers and people with flushing responses [60–62]. However, taking liver holidays was associated with a lower risk of cancer and CVD mortality in men [60]. The Miyagi cohort study estimated that 17.9% of all cancers affecting men could be omitted by avoiding excessive drinking [63]. In the JPHC Study, light to moderate drinking protected against CHD and ischemic stroke risk, but excessive drinking increased hemorrhagic and ischemic strokes [64–66]. In addition, excessive drinking was associated with an elevated risk of AF in the Suita Study [23].

Similar to cancer and CVD, the Okayama Study showed that light drinking could reduce dementia risk [67], while the Hisayama Study showed that excessive drinking increased that risk [68]. Further, a systematic review of 7 Japanese cohort studies showed that excessive drinking could increase the risk of diabetes, while the protective effect of light drinking was inconclusive [69].

Our third health tip is to refrain from excessive drinking. It should be kept in mind that alcohol dependence is a disorder that cannot get cured without proper medical consultation. Middle-aged men should limit alcohol intake to 20 g/day. Women, older adults, and those with flushing responses or liver disorders should consume lower amounts [6, 60]. Taking liver holidays is warranted [60]. Generally, drinking should be avoided before bathing, PA, or work, and no alcohol is allowed during pregnancy and lactation. Those who do not drink should be advised against starting. On the level of health policies, raising alcohol taxes, reducing alcohol sales, and limiting the access of minors to alcohol should be urged.

### 4. Reduce fried foods and sugary soft drinks

Japanese food is widely thought to be healthy, but it includes some unhealthy foods especially deep-oil-fried foods such as tempura (deep-fried mixture of flour, egg, and meat or fish), croquette (deep-fried dumpling of a thick binder and mashed potato), tonkatsu (breaded pork cutlets), and karaage (Japanese fried chicken) [70, 71]. The National Health and Nutritional Surveys have shown a continuous increase in the consumption of fried foods in Japan [72]. Besides, the intake of sugary soft drinks has been increasing along with Westernizing the Japanese dietary patterns [73, 74].

The consumption of deep-fried food was shown to have negative health impacts. For example, the Fukuoka Prefecture cohort study indicated that stomach cancer mortality was tripled among those who reported frequent use of cooking oil [75]. A tendency toward increased stomach cancer mortality with fried foods was also shown in a cohort study including rural residents from Aichi Prefecture [76].

On the other hand, consuming sugary soft drinks was linked to cancer and CVD risk. Women consuming sugary soft drinks in the JPHC Study had a higher risk of colon, kidney, and bladder cancers [77, 78] and ischemic stroke [79]. Besides, the JPHC Study showed that sugary soft drinks were associated with increased all-cause mortality by 17%, CVD mortality by 26%, and diabetes incidence by 79% [80, 81].

Our fourth health step is to reduce fried food consumption and avoid drinking sugary soft drinks. We think that planning meals ahead is the best way to improve dietary patterns. These patterns should include fewer fried foods and no sugary soft drinks. Besides, do not add sugar to tea, coffee, and fresh juice. We recommend replacing sugary soft drinks with other drinks such as Japanese or Chinese tea (green tea, oolong tea, and chrysanthemum tea).

### 5. Reduce salt intake

The traditional Japanese dietary pattern tends to be salty [82]; however, the National Health and Nutrition Surveys reported a decreasing trend in salt intake between 1995 and 2016, but it stands for almost twice the recommended levels [83].

Salt intake has several adverse health impacts. The JACC Study showed that participants who preferred salty food were more likely to develop stomach cancer, and compared to people in the lowest quantile of salt intake, those in the third, fourth, and fifth quantiles showed a higher risk of stomach cancer by 36%, 29%, and 51% [84]. The JPHC Study showed similar associations between salt or salty food intake and stomach cancer incidence and mortality [85, 86]. Salt intake was associated with increased CVD and stroke risk in the JPHC Study [87] and stroke mortality in the Takayama Study [88]. The NIPPON DATA80 showed that increased dietary Na–K ratio was associated with increased mortality due to all-cause, CVD, and ischemic and hemorrhagic strokes [89]. Also, the increase in household salt intake in the NIPPON DATA80 was modestly associated with increased mortality due to all-cause, CVD, CHD, and stroke [90], while adherence to a healthy reduced-salt Japanese diet was associated with reductions in all-cause and CVD mortality [91]. A significant association between salt intake and blood pressure was also reported in the NIPPON DATA80 Study [92].

The fifth health tip refers to the importance of cutting salt in food and reducing salty food intake. The target of salt consumption for the general population in Japan should be <7.5 g/day for men and <6.5 g/day for women [6]. The JSH recommended lower amounts to prevent hypertension and for those with hypertension to control their increased blood pressure (<6 g/day). The European guidelines recommended even lower levels of salt consumption (<5 gm/day). The traditional Japanese diet is considered healthy despite containing a high salt component. This could partly explain why the standard salt consumption level was set higher than that in the European guidelines. However, the saying that it is acceptable for the Japanese diet to be salty as long as it is healthy lacks evidence. Minimizing salt consumption can be achieved by decreasing the intake of salty food, checking food labels for salt content, using spices, herbs, and citrus juice in place of salt to add flavor, and taking salt and salty sauces off the dining table. It is advised to avoid drinking noodle soup, limit the amount of miso soup, a salty soup made of fermented soya bean paste, to one cup/day, consume less tsukemono (traditional Japanese pickled vegetables), minimize the usage of salty soy sauce or use low-or zero-salt soy sauce instead, and avoid processed meat. On the national policy level, people should be educated about the harms of excessive salt intake, salt reduction promotion should be included as a part of food education at schools, and food companies and restaurants should be encouraged to reduce the salt content in their products.

### 6. Eat more soy food

Soy intake is a feature of the Japanese diet. The Japanese people consume significantly more soy products than their counterparts in Western communities [93, 94]. The major sources of soy in the Japanese diet are miso, natto, tofu, soybeans, kinako (powdered dried soybeans), and soymilk. Miso and natto are fermented forms of soy [95, 96].

Soy foods have potential protective effects against cancer and CVD. The Takayama showed that soy intake was associated with decreased stomach cancer, and the protective effect was more prominent with non-fermented than fermented soy products [97, 98]. A systematic review of 5 cohort studies and 6 case-control studies investigating Japanese populations concluded that soy intake was likely to reduce the risk of breast cancer in women [99]. Fermented soy in the JPHC Study was associated with the decreased risk of nonlocalized breast cancer, CVD, and stroke [100, 101] and mortality due to all-cause and CVD [102]. An earlier study from the JPHC Study indicated that women consuming soy food 5 times/week had lower risks of cerebral infarctions, myocardial infarctions, and CVD mortality by 36%, 45%, and 69%, and an inverse association between isoflavone intake and risk of cerebral and myocardial infarctions was especially observed among postmenopausal women [103]. The Takayama Study showed that the intakes of the highest quartiles of soy protein intake were associated with a 25% reduction in stroke mortality [104].

In addition to preventing cancer and CVD, adherence to a dietary pattern with a high intake of soy food in the Hisayama Study was associated with a 34% decrease in the risk of dementia [105]. In the JPHC Study and the Takayama Study, women consuming soy products were less likely to develop diabetes [106, 107].

Given the health benefits of soy foods, our sixth health tip is to consume more soy products. Increasing soy intake can be achieved by frequently adding natto, boiled beans, and tofu as side dishes, drinking soymilk, using soy flour or butter for baking, and cooking with low-or zero-salt miso sauce.

### 7. Consume enough fiber every day

There are two major types of fibers included in food: soluble and insoluble. In the Japanese diet, seaweed and fruits are the major sources of soluble fiber, while rice, mushroom, oatmeal, whole grains, vegetables, beans, nuts, and legumes are the major sources of insoluble fiber. Insufficient fiber consumption is a challenge to health promotion in Japan as fiber intake has been declining since 1952, especially among younger generations. In particular, there is a shortage in consuming water-soluble dietary fiber [108].

Dietary fibers have several health benefits. The JPHC Study showed a tendency toward an inverse association between cereal fiber intake and the risk of stomach cancer in men [109], reduced risk of advanced prostate cancer among men consuming high total and insoluble fiber [110], inverse trends between total fiber and breast cancer risk [111], a negative association between total fiber intake and CVD incidence in nonsmokers [112], and reduced CVD and all-cause mortality among people consuming fibers from fruits, vegetables, and beans [113]. The NIPPON DATA80 indicated that the highest quartile of total fiber intake was associated with a 36% reduction in CVD mortality among men and a 39% reduction in stroke mortality among women [114]. The JACC Study revealed that fiber intake, soluble and insoluble, almost halved the risk of CVD mortality and reduced CHD mortality by 20%, and fibers from fruits and cereals had more cardiovascular protective properties [115].

Besides, the Hisayama Study showed that high dietary fiber intake, soluble and insoluble, was associated with a decreased incidence of diabetes [116]. Increased dietary fiber in the Fukuoka Diabetes Registry was associated with glycemic control and a low incidence of end-stage renal disease among diabetics [117, 118]. The Furukawa Nutrition and Health Study showed an inverse association between total fiber intake and depressive symptoms [119].

The seventh health tip is to eat more fibers. It is advised that men and women (18–64 years) should consume a minimum of 21 and 18 g/day of fibers, while older men and women should consume a minimum of 20 and 17 g/day of fibers [6]. Adding vegetables to meals, snacking on fruits, adding pulses like lentils and beans to salads and curries, and replacing refined grains (wheat and rice) with unrefined ones (whole wheat bread, brown rice, oatmeal, barley, bran cereal, and quinoa) could help get enough healthy fibers every day.

### 8. Include fruits and vegetables in meals

While consuming fruits and vegetables is highly recommended, it seems that the prevalence of those who intake enough vegetables and fruits in Japan, especially among adolescents, is noticeably low [120].

Fruits and vegetables were shown to improve health in Japanese literature. The JPHC Study indicated that eating vegetables and fruits was associated with a lower risk of stomach and esophageal cancers [121, 122], and fruit consumers, especially non-smokers, had a reduced risk of pancreatic cancer [123]. A pooled analysis of 4 Japanese population-based cohort studies concluded that fruit consumption was associated with a decreased risk of lung cancer among men, while vegetable consumption was associated with a decreased risk of stomach cancer [124, 125]. Low CVD incidence and mortality risk was shown among fruit and vegetable consumers in the JPHC Study, JACC Study, and NIPPON DATA80 [126–128]. Serum vitamin C concentration among people registered in the Shibata Study was inversely associated with the risk of cerebral infarction and hemorrhagic stroke [129].

Further, the Hisayama Study showed that vegetable-rich diets were associated with a lower dementia risk [105], while The SONIC study showed that a plant pattern diet rich in fruits and vegetables was associated with better cognitive functions among Japanese older adults [130]. Moreover, vegetable intake in the Fukushima Health Management Survey was associated with a lower risk of CKD [131].

The eighth health tip is to eat more fruits and vegetables. The daily recommended amounts of fruit and vegetables are ≥200 g and ≥350 g [6]. This could be achieved by putting fruits and vegetables on the grocery list, adding fruits and vegetables to dishes, eating green salad with meals, adding a side dish of raw, boiled, or steamed vegetables with each meal, and snacking on fruits.

### 9. Consume fish regularly

Fish meals are characteristic of the Japanese dietary pattern and are related to several favorable health impacts [93, 132]. Fish is rich in omega-3 polyunsaturated fatty acids (PUFAs) that have anticarcinogenic properties [133] and confer many benefits for cardiovascular health [134].

The Miyako Study showed that high fish intake was associated with an 88% reduction in prostate cancer mortality [135]. The intake of the highest quartile of marine omega-3 PUFAs was associated with a 30% reduction in pancreatic cancer risk in the JPHC Study [136]. A cohort study including residents of rural areas in Aichi Prefecture showed that eating fish and shellfish 3 times/week was associated with a 68% reduction in incident lung cancer [137]. Also, eating fish 3–4 times/week in the JACC Study was associated with reduced liver cancer mortality in older men and women by 66% and 71% [138]. In the JPHC Study, eating fish 8 times/week or seaweed every day was associated with a decreased CHD risk [139, 140]. In the JACC Study, the intakes of the highest quantiles of fish and omega-3 PUFAs were shown to be associated with reductions in CVD and heart failure mortality [141].

In addition, fish intake in the JPHC Study was associated with a reduced risk of dementia [142], major depressive disorders [143], and diabetes [144]. Alike, the Ohsaki Study showed a dose-response association between fish intake and incident dementia [145].

Our ninth health tip is to consume more fish. Replacing meat and chicken with fish or seafood as a major source of animal protein is recommended. For those who find it difficult to cook fish, consuming commercially available low-salt tuna and mackerel cans may help. Further, providing fish at lower prices can encourage people to change their dietary habits. Raising people’s awareness about the health benefits of fish should be promoted on a broader scale.

### 10. Maintain proper body weight

Unlike the widely known definitions for overweight and obesity as body mass index (BMI) 25–29.9 and ≥30 kg/m^2^, respectively, the BMI cut-off for obesity in Japan is 25 kg/m^2^. The prevalence of obesity has been increasing in Japan, yet with much lower paces than in Western countries [146].

Obesity is a well-known risk factor for cancer and CVD. The JACC Study showed that the higher BMI categories were associated with increased obesity-related cancer mortality in women, incident CHD in men and women, and CVD mortality in men and women [147, 148]. In the JPHC Study, the BMI category ≥30 kg/m^2^ among men increased cancer risk by 22% [16], while cardioembolic stroke risk more than doubled [149]. The Ohsaki Study showed increased CVD, CHD, and stroke mortality in obese people [150]. Further, the JMS study reported an increased risk of all-cause, cancer, and CVD mortality among obese people, even those who were metabolically healthy [151]. Abdominal obesity was also shown to be associated with CVD risk in the Suita Study [152]. A recent pooled analysis of 10 Japanese cohort studies showed that both obesity and abdominal obesity were associated with increased CVD risk [153]. Obesity was also associated with incident diabetes in the Ibaraki Prefectural Health Study [154] and CKD risk in a cohort study including factory workers from Toyama Prefecture [155].

Like obesity, being underweight was documented in Japanese epidemiological literature as a risk factor for cancer and CVD mortality [16, 147, 148, 150, 151, 156]. The JAGES revealed a higher dementia incidence among underweight older adults [157]. Being underweight during adolescence was associated with adult-onset diabetes among women in the Japan Nurses’ Health Study [158]. Besides, excessive weight change was shown to be related to all-cause, cancer, and CVD mortality [159–163]. However, these studies did not show whether weight change was intentional or not.

Our tenth health tip is to maintain proper body weight and avoid gaining or losing excessive weight. Eating regular meals with reasonable portions, consuming more vegetables and fruits, practicing PA, eating high fiber food, and avoiding junk food can efficiently help in maintaining a healthy weight. Consulting health specialists before managing to gain or lose weight is warranted. Further, while having a BMI <25 kg/m^2^ is the standard, abdominal obesity could contribute to unfavorable health outcomes even with a normal BMI [164, 165].

## Perspectives

We believe that the LHS10 items can be good material for health guidance whether for the general population or patients with different chronic diseases. Also, it could help individualize health messages and promote people’s health based on their medical condition and lifestyle expectations. When people or patients realize how much their daily behaviors agree with the ideal lifestyle, they can take steps towards improving their lifestyle.

Of note, applying the LHS10 should not be detached from improving the overall socioeconomic status and environment and finding solutions to the income-based inequality in healthcare access and check-up participation [166–168]. There is a solid link between poor economic status and unfavorable lifestyle and health behaviors and consequently higher levels of morbidities and mortalities from chronic diseases [169]. Alike, poor social environment and unsanitary place of residence could negatively affect lifestyle and have lifelong health implications [169].

Since similar health items were suggested by international literature to promote a healthy lifestyle, the LHS10 can apply to non-Japanese populations. It can be said that the LHS10 items are universal and do not materially change across races and cultures [9].

Further, it should be noted that the LHS10 items and related health tips should be updated regularly after considering emerging epidemiological evidence from prospective cohort studies and non-pharmacological intervention trials.

## Conclusions

The LHS10 included avoiding smoking and secondhand smoke exposure, practicing PA, refraining from excessive alcohol drinking, reducing fried food and sugary soft drink consumption, cutting salt in food, consuming more soy products, fibers, vegetables, fruits, and fish, and maintaining proper body weight. This review provided substantial epidemiological evidence from the Japanese literature showing the protective effects of the LHS10 items against the risk of cancer, CVD, and other chronic diseases.

## Abbreviations

AF: Atrial Fibrillation
BMI: Body Mass Index
CHD: Coronary Heart Disease
CKD: Chronic Kidney Disease
CVD: Cardiovascular Disease
JACC: Japan Collaborative Cohort Study
JAGES: Japan Gerontological Evaluation Study
JMS: Jichi Medical School Study
JPHC: Japan Public Health Center-based Prospective Study
J-ECOH: Japan Epidemiology Collaboration on Occupational Health
LHS10: Lifelong Health Support 10
NIPPON DATA80: National Integrated Project for Prospective Observation of Non-communicable Disease And its Trends in the Aged
PA: Physical Activity
PUFAs: Polyunsaturated Fatty Acids
WHO: World Health Organization

## Supplementary information

**Additional file 1:** Components of the Lifelong Health Support 10.

## Declarations

### Data availability

Non-applicable.
