# Supplementary figures and images for: The Lifelong Health Support 10: a Japanese prescription for a long and healthy life
Source: Environ Health Prev Med. 2022 Jun 9;27:23. doi: 10.1265/ehpm.22-00085 (PMC9251624; doi:10.1265/ehpm.22-00085)

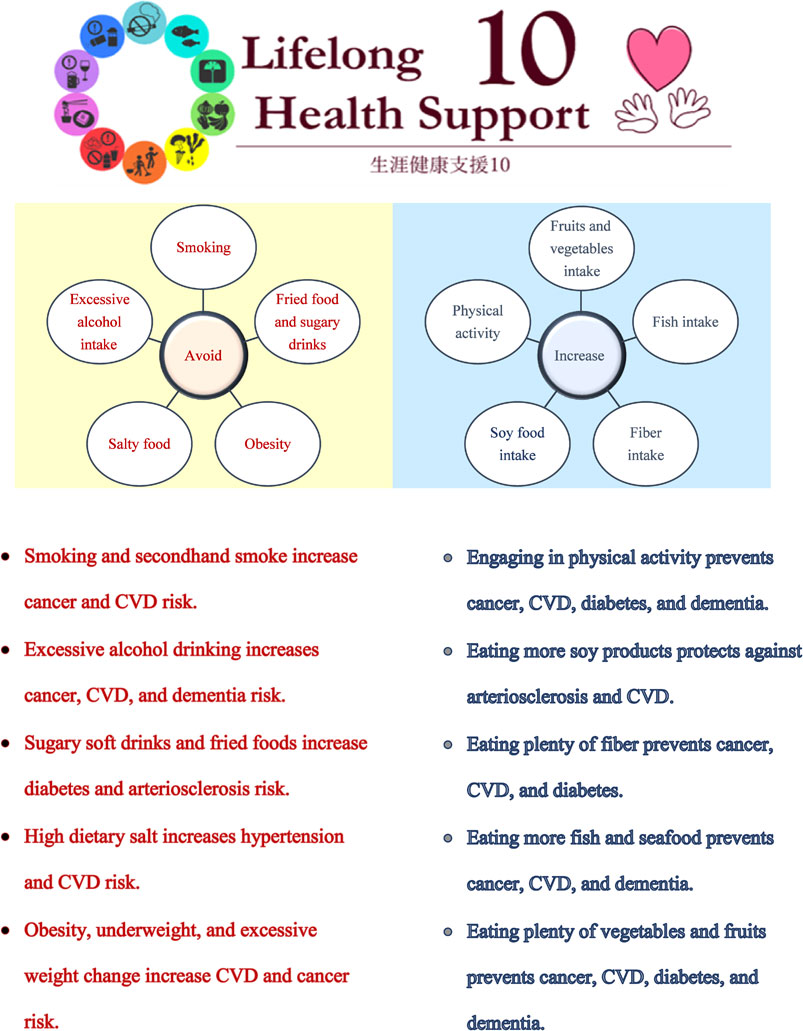

Supplement: Supplementary file 1 — Additional file 1: Components of the Lifelong Health Support 10. [file ehpm-27-023-s001.jpg]
